# A reappraisal of transcriptional regulation by NR5A1 and beta-catenin in adrenocortical carcinoma

**DOI:** 10.3389/fendo.2023.1303332

**Published:** 2023-12-08

**Authors:** Enzo Lalli

**Affiliations:** ^1^ Institut de Pharmacologie Moléculaire et Cellulaire CNRS UMR 7275, Valbonne, France; ^2^ Université Côte d’Azur, Valbonne, France; ^3^ Inserm, Valbonne, France

**Keywords:** adrenocortical carcinoma, beta-catenin, nuclear receptors, transcriptional regulation, genomics

## Abstract

**Background:**

Overexpression of the transcription factor NR5A1 and constitutive activation of canonical Wnt signalling leading to nuclear translocation of beta-catenin are hallmarks of malignancy in adrenocortical carcinoma (ACC). Based on the analysis of genomic profiles in H295R ACC cells, Mohan et al. (*Cancer Res*. 2023; 83: 2123-2141) recently suggested that a major determinant driving proliferation and differentiation in malignant ACC is the interaction of NR5A1 and beta-catenin on chromatin to regulate gene expression.

**Methods:**

I reanalyzed the same set of data generated by Mohan et al. and other published data of knockdown-validated NR5A1 and beta-catenin target genes,

**Results:**

Beta-catenin is mainly found in association to canonical T cell factor/lymphoid enhancer factor (TCF/LEF) motifs in genomic DNA. NR5A1 and beta-catenin regulate distinct target gene sets in ACC cells.

**Conclusion:**

Overall, my analysis suggests a model where NR5A1 overexpression and beta-catenin activation principally act independently, rather than functionally interacting, to drive ACC malignancy.

## 1 Introduction

Recent studies have allowed to make much progress in our understanding of the molecular and genomic determinants implicated in the pathogenesis of adrenocortical carcinoma (ACC), a rare endocrine malignancy. Among those factors, a key role is played by overexpression of the transcription factor Steroidogenic Factor-1/NR5A1 and activation of canonical Wnt signalling. NR5A1 overexpression is a common finding in paediatric ACC (1) and is a marker of malignancy in adults (2). In ACC cells, NR5A1 overexpression is sufficient to regulate the expression of a set of genes linked to malignancy in a dosage-dependent fashion (3–8). On the other hand, somatic mutations of *Catenin beta 1* (*CTNNB1)* or other genes leading to constitutive activation of the canonical Wnt pathway are present in about 30% of ACC, alone or in combination with other genomic alterations (9–11). Beta-catenin activation is associated to poor outcome in ACC (12). Activated beta-catenin translocates to the nucleus, where it regulates gene expression mainly by association with the TCF/LEF family of transcription factors, even if its interaction with other classes of transcription factors has been described, including nuclear receptors (13–15). A number of target genes for activated beta-catenin has been described in the H295R ACC cell line (where beta-catenin is constitutively activated due to a *CTNNB1* mutation) after selective downregulation of beta catenin by expression of an inducible shRNA (16).

Little is known about the potential interplay of those factors in driving ACC malignancy. Based on the analysis of genomic profiles in H295R cells, a recent study suggested that a major determinant driving proliferation and differentiation in malignant ACC is the interaction of NR5A1 and beta-catenin on chromatin to regulate gene expression (17). However, by the analysis of the data generated by Mohan et al. and of published data of knockdown-validated NR5A1 and beta-catenin target genes, here I show that beta-catenin is mainly found in association to canonical TCF/LEF motifs in genomic DNA and that NR5A1 and beta-catenin regulate distinct target gene sets in H295R ACC cells. These results are strongly suggestive that NR5A1 and beta-catenin act independently, rather than functionally interacting, to shape the malignant phenotype of ACC cells.

## 2 Materials and methods

### 2.1 ChIP-seq data analysis

NR5A1 (basal; SRR19503712), beta-catenin (basal; SRR19503710) ChIP-seq and input DNA (SRR19503702) fastq files from the study by Mohan et al. (17) were retrieved from the SRA database (https://www.ncbi.nlm.nih.gov/sra). All data analyses were performed in the Galaxy server (https://usegalaxy.eu) (18). After quality control and filtering below 20 cut-off value, reads were mapped on the human genome (version hg38) using Bowtie2 and default values. For both NR5A1 and beta-catenin ChIP-seq samples narrow peaks were called from bam files using MACS2 with the following parameters: control file, input DNA; effective genome size, 2,700,000,000; build model; lower mfold bound, 5; upper mfold bound, 50; band width for picking regions to compute fragment size, 300; peak detection based on 0.05 q-value. After excluding ENCODE blacklisted regions (ENCFF356LFX), 47,071 peak regions were obtained for the NR5A1 sample and 1,055 for the beta-catenin sample. Overlap between those two datasets (979 regions) was calculated using the Intersect interval tool. The MEME suite (https://meme-suite.org/meme) (19) was used to analyze DNA motifs present in the overlapping NR5A1 – beta-catenin ChIP peaks. After motif analysis by MEME-ChIP, the presence of the TCF7L2 (MA0523.1 in JASPAR) and NR5A2 (MA0505.2 in JASPAR) motifs in those sequences was searched by the FIMO tool.

### 2.2 Gene expression analysis

The lists of genes significantly differentially expressed after NR5A1 (5, 20, 21) and beta-catenin (16) knockdown in H295R cells were compared and results visualized using jvenn (22). Gene Ontology analysis of NR5A1 and beta-catenin target genes was performed using Metascape (23).

## 3 Results

### 3.1 Beta-catenin genomic binding sites overlapping with NR5A1 binding sites in H295R cells are enriched with TCF7L2 motifs

**Figure 1:**
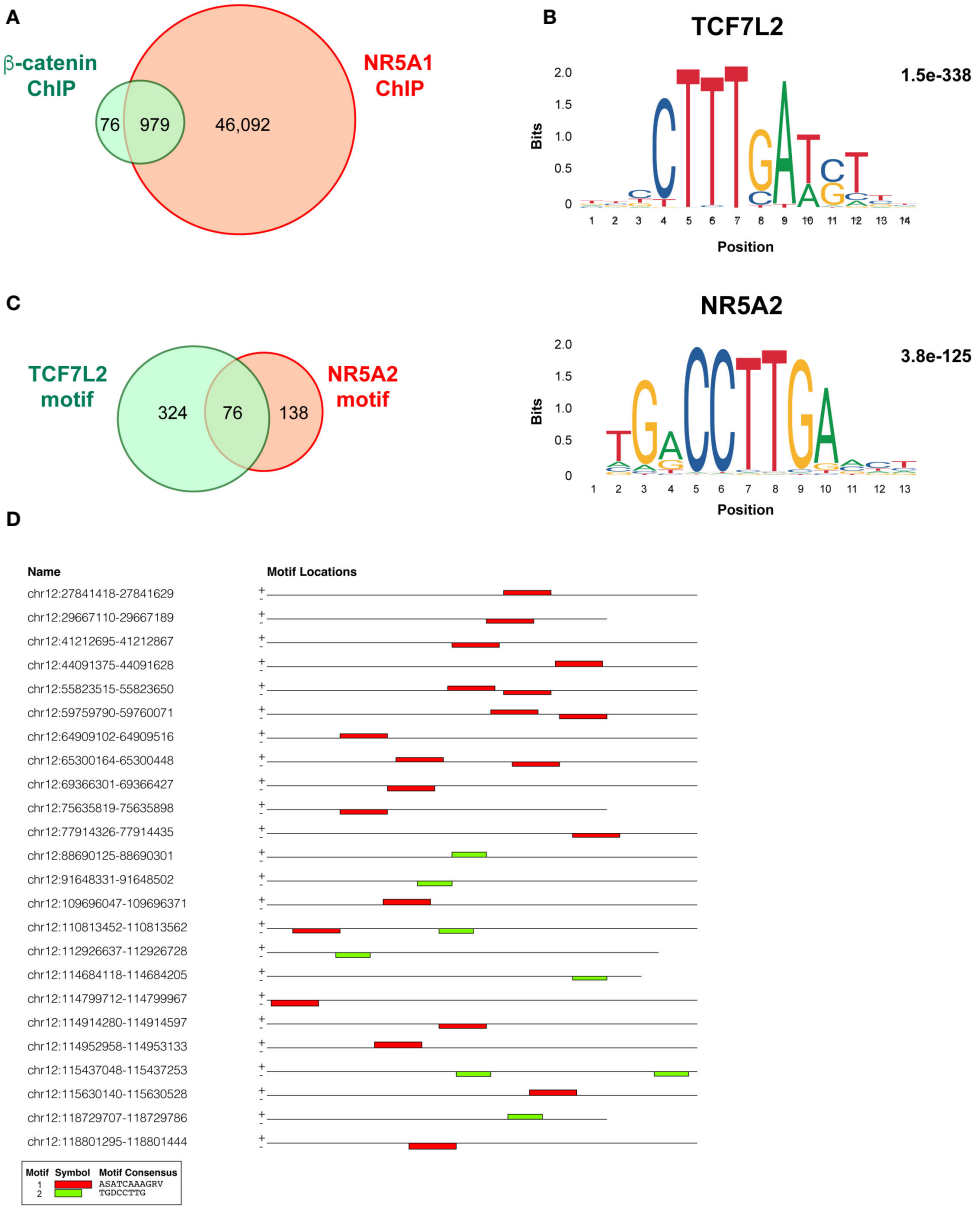
Overlap and motifs in NR5A1 and beta-catenin genomic binding sites in H295R ACC cells. **(A)** Overlap between NR5A1 and beta-catenin ChIP peaks. **(B)** DNA motifs enriched in the 979 NR5A1 – beta-catenin intersect peaks and statistical significance value as calculated by MEME. **(C)** Abundance of TCF7L2, NR5A2 or both motifs in the 979 NR5A1 – beta-catenin intersect peaks, as calculated by FIMO. **(D)** Examples of the presence of various combinations of the TCF7L2, NR5A2 or both motifs in the sequences of NR5A1 – beta-catenin intersect peaks.

My analysis performed using MACS2 software on the ChIP-seq data from the Mohan et al.’s study (17) revealed a total of 47,071 genomic binding sites for NR5A1 and 1,055 binding sites for beta-catenin within H295R cells. Notably, there were 979 binding sites overlapping binding sites (**Figure 1A**, **Supplementary Table S1** for details). This is a much smaller figure than the number of overlapping NR5A1 – beta-catenin binding sites reported by Mohan et al. (3,559). TCF7L2 and NR5A2 motifs were significantly enriched in beta-catenin binding sites (1.5e-338 and 3.8e-125, respectively) (**Figure 1B**). Even if differences in data analysis methods and thresholds used may account in part for the discrepancies of my analysis with what reported by Mohan et al. (17), it is remarkable that out of the 979 NR5A1 – beta-catenin overlapping peaks, 400 displayed one or more TCF7L2 motifs, associated or not to a NR5A2 motif, while only 214 NR5A1/beta-catenin intersect ChIP peaks displayed a NR5A2 motif (76 also harbouring one or multiple TCF7L2 motif). In addition, 441 NR5A1/beta-catenin intersect ChIP peaks harboured neither motif (**Figure 1C**, **Supplementary Table S2**). These data strongly suggest that beta-catenin predominantly interacts with cognate TCF motifs even within overlapping NR5A1 – beta-catenin ChIP peaks. Examples of adjacent TCF7L2 and NR5A2 motifs within an overlapping NR5A1 – beta-catenin genomic binding site are shown in **Figure 1D**. NR5A1/beta-catenin intersect ChIP peaks harbouring TCF7L2, NR5A2 or both motifs have a similar genomic distribution in relationship to gene elements (**Figure S1**).

### 3.2 Little overlap among NR5A1 and beta-catenin target genes in H295R cells

**Figure 2:**
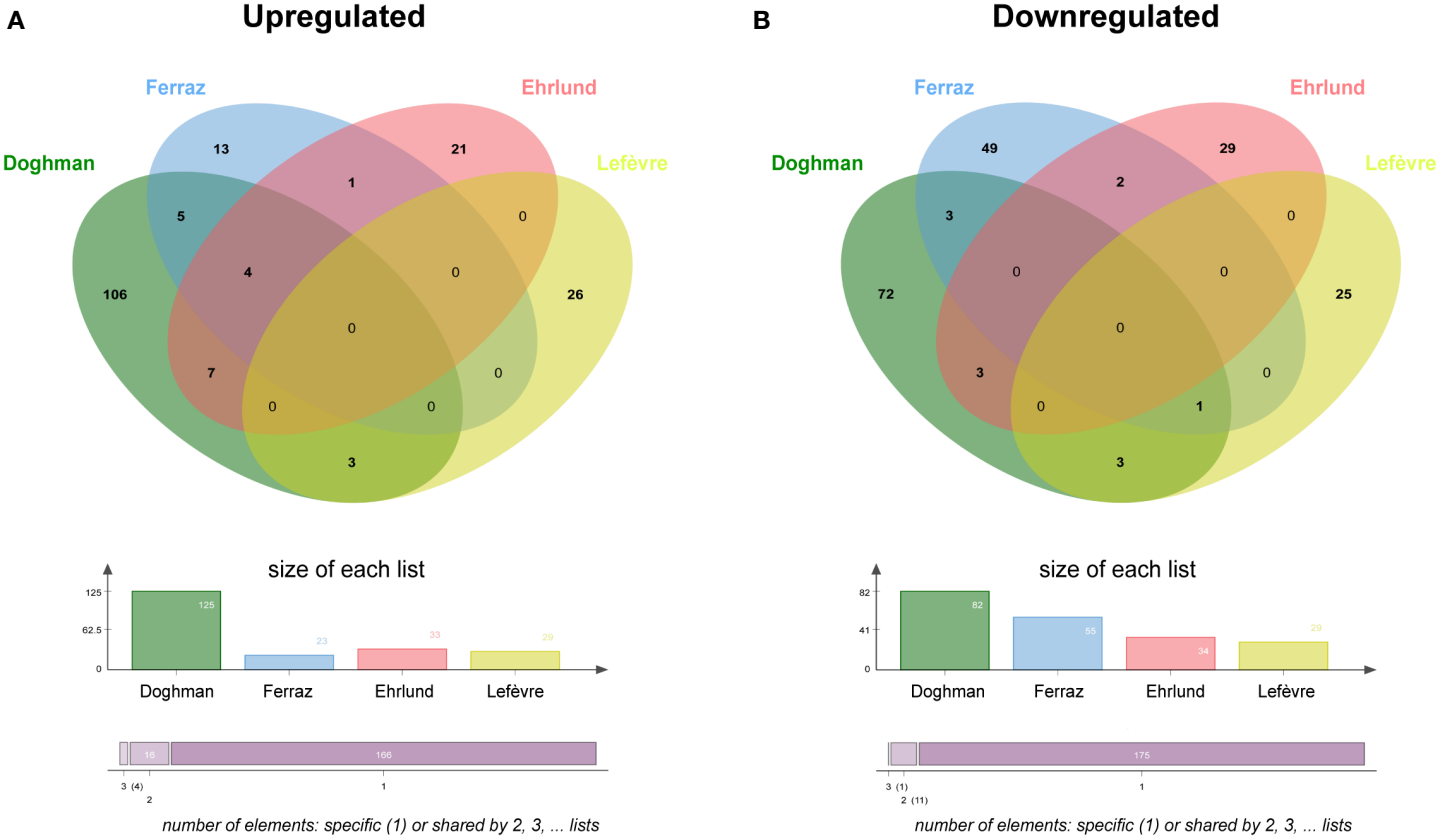
NR5A1 and beta-catenin – regulated genes in H295R cells. **(A)** Overlap among genes upregulated (downregulated after knockdown) by NR5A1 and beta-catenin, respectively. **(B)** Overlap among genes downregulated (upregulated after knockdown) by NR5A1 and beta-catenin, respectively. NR5A1 data are derived from three different datasets (5, 20, 21). Beta-catenin data from Lefèvre et al. (16).

Binding to genomic DNA does not represent evidence for gene regulation unless complemented with functional data. To characterize the crosstalk of NR5A1 and beta-catenin on the regulation of gene expression programs in H295R cells, I have compared the published datasets of both NR5A – regulated (5, 20, 21) and beta-catenin – regulated (16) genes in this cell line. Enriched Gene Ontology categories for genes regulated either positively or negatively by NR5A1 and beta-catenin are shown in **Figure S2**. Prominent enriched categories are genes involved in steroidogenesis for NR5A1 – positively regulated genes, locomotion for NR5A1 – negatively regulated genes, Wnt signalling for beta-catenin – positively regulated genes and regulation of actin cytoskeleton for beta-catenin – negatively regulated genes. Out of 29 genes positively regulated by beta-catenin, only 3 (*CADPS*, *GRPR* and *ISM1*) are in common with genes positively regulated by NR5A1 in at least one of those datasets (**Figure 2A**, **Supplementary Table S3**). On the other hand, out of 29 genes negatively regulated by beta-catenin, only 4 genes (*ITGA8*, *JAG1*, *OTULINL* and *LXN*) were commonly downregulated by NR5A1 and beta-catenin in at least one dataset (**Figure 2B**, **Supplementary Table S3**).

## 4 Discussion

Previous studies have reported physical and functional interaction between NR5A1 and beta-catenin to regulate transcription of specific target genes (13, 24, 25). However, the study by Mohan et al. (17) has been the first to investigate the localization of both NR5A1 and beta-catenin binding sites in the chromatin of H295R ACC cells at the genome-wide scale. Those authors concluded that the interaction between those factors is a major determinant driving proliferation and differentiation in malignant ACC.

**Figure 3:**
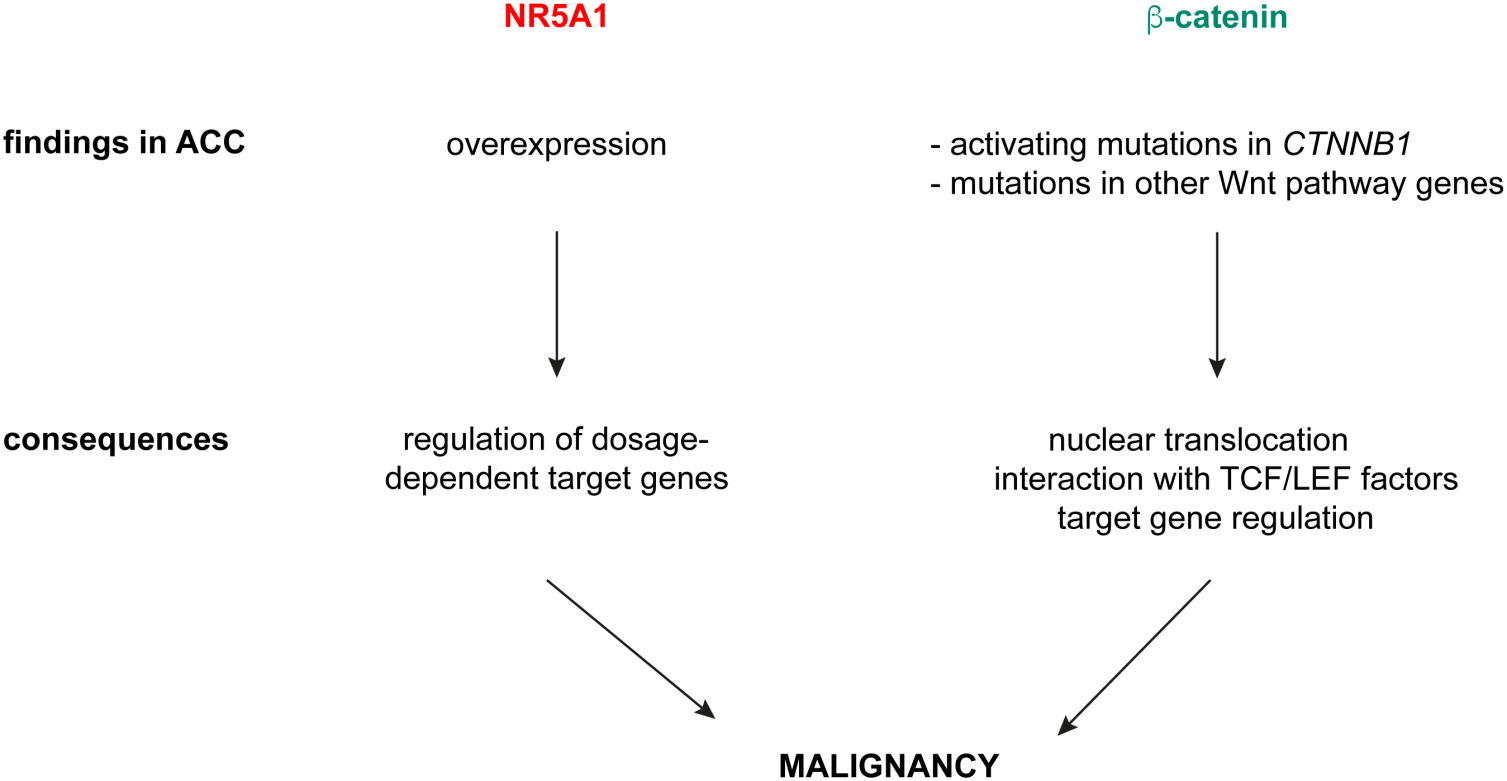
A model for NR5A1 and beta-catenin in driving ACC malignancy. NR5A1 is found overexpressed in ACC, with consequent transcriptional regulation of dosage-dependent target genes involved in several aspects of the malignant phenotype. Beta-catenin activation in ACC is caused by activating mutations of *CTNNB1* or inactivating mutations in negative regulators of canonical Wnt signalling (*ZNRF3*, *APC*). Those produce nuclear translocation of beta-catenin, which interacts preferentially with TCF/LEF transcription factors and regulates target gene expression.

I have shown here that overlapping NR5A1 – beta-catenin genomic binding sites in H295R cells contain in large proportion canonical TCF/LEF motifs, alone or in combination with nuclear receptor half-sites known as binding motifs for NR5A1/NR5A2. This finding strongly suggests that interaction of beta-catenin with cognate TCF/LEF transcription factors are dominant to shape the transcriptional profiles of its target genes in ACC cells and are consistent with the results by Schuijers et al. which showed that beta-catenin acts nearly exclusively through interaction with TCF/LEF in colon cancer cells (15). Furthermore, target gene sets regulated by NR5A1 and beta-catenin in H295R cells are divergent (**Figure 2**). Overall, in contrast to the conclusions by the Mohan et al. article (17), these data provide compelling evidence that in ACC cells NR5A1 and beta-catenin, which are both relevant factors driving tumour malignancy, regulate mostly distinct gene expression programs through different mechanisms. We have demonstrated that NR5A1 overexpression in ACC cells regulates the expression of both positive and negative dosage-dependent target genes which are directly implicated in shaping the malignant tumour phenotype (3–8). On the other hand, activating mutations in *CTNNB1* and other genetic alterations in canonical Wnt pathway components in ACC induce nuclear translocation of beta-catenin and transcriptional regulation of genes involved in cell proliferation, apoptosis and invasion, being also associated to immune cell exclusion from the tumour (16, 26–31). Overall, these data suggest a model where NR5A1 overexpression and beta-catenin activation principally act in parallel, rather than functionally interacting, to drive ACC malignancy (**Figure 3**). Interestingly, both factors can be targeted by small-molecule inhibitors (26, 32, 33) some of which have already reached the clinical stage. Combined inhibition of both NR5A1 and beta-catenin can then be a promising innovative therapeutic strategy for ACC.

## Data availability statement

Publicly available datasets were analyzed in this study. This data can be found here: SRR19503702, SRR19503710, SRR19503712 from the SRA database: https://www.ncbi.nlm.nih.gov/sra.

## Author contributions

EL: Formal analysis, Funding acquisition, Methodology, Writing – original draft, Writing – review & editing.

## References

[1] Pianovski MA Cavalli LR Figueiredo BC Santos SC Doghman M Ribeiro RC. SF-1 overexpression in childhood adrenocortical tumours. Eur J Cancer (2006) 42:1040–3. doi: 10.1016/j.ejca.2006.01.022 16574405

[2] Sbiera S Schmull S Assie G Voelker HU Kraus L Beyer M. High diagnostic and prognostic value of steroidogenic factor-1 expression in adrenal tumors. J Clin Endocrinol Metab (2010) 95:E161–71. doi: 10.1210/jc.2010-0653 20660055

[3] Doghman M Karpova T Rodrigues GA Arhatte M De Moura J Cavalli LR. Increased Steroidogenic Factor-1 dosage triggers adrenocortical cell proliferation and cancer. Mol Endocrinol (2007) 21:2968–87. doi: 10.1210/me.2007-0120 17761949

[4] Doghman M Arhatte M Thibout H Rodrigues G De Moura J Grosso S. Nephroblastoma overexpressed/cysteine-rich protein 61/connective tissue growth factor/nephroblastoma overexpressed gene-3 (NOV/CCN3), a selective adrenocortical cell proapoptotic factor, is down-regulated in childhood adrenocortical tumors. J Clin Endocrinol Metab (2007) 92:3253–60. doi: 10.1210/jc.2007-0342 17566092

[5] Doghman M Figueiredo BC Volante M Papotti M Lalli E. Integrative analysis of SF-1 transcription factor dosage impact on genome-wide binding and gene expression regulation. Nucl Acids Res (2013) 41:8896–907. doi: 10.1093/nar/gkt658 PMC3799431 23907384

[6] Doghman-Bouguerra M Granatiero V Sbiera S Sbiera I Lacas-Gervais S Brau F. FATE1 antagonizes calcium- and drug-induced apoptosis by uncoupling ER and mitochondria. EMBO Rep (2016) 17:1264–80. doi: 10.15252/embr.201541504 PMC5007562 27402544

[7] Ruggiero C Doghman-Bouguerra M Sbiera S Sbiera I Parsons M Ragazzon B. Dosage-dependent regulation of VAV2 expression by Steroidogenic Factor-1 drives adrenocortical carcinoma cell invasion. Sci Signal (2017) 10:eaal2464. doi: 10.1126/scisignal.aal2464 28270555

[8] Relav L Doghman-Bouguerra M Ruggiero C Muzzi JCD Figueiredo BC Lalli E. Steroidogenic Factor 1, a Goldilocks transcription factor from adrenocortical organogenesis to Malignancy. Int J Mol Sci (2023) 24:3585. doi: 10.3390/ijms24043585 36835002 PMC9959402

[9] El Wakil A. Lalli E 2011 The Wnt/beta-catenin pathway in adrenocortical development and cancer. Mol Cell Endocrinol (2011) 332:32–7. doi: 10.1016/j.mce.2010.11.014 21094679

[10] Assié G Letouzé E Fassnacht M Jouinot A Luscap W Barreau O. Integrated genomic characterization of adrenocortical carcinoma. Nat Genet (2014) 46:607–12. doi: 10.1038/ng.2953 24747642

[11] Lippert J Appenzeller S Liang R Sbiera S Kircher S Altieri B. Targeted molecular analysis in adrenocortical carcinomas: a strategy toward improved personalized prognostication. J Clin Endocrinol Metab (2018) 103:4511–23. doi: 10.1210/jc.2018-01348 30113656

[12] Gaujoux S Grabar S Fassnacht M Ragazzon B Launay P Libé R. β-catenin activation is associated with specific clinical and pathologic characteristics and a poor outcome in adrenocortical carcinoma. Clin Cancer Res (2011) 17:328–36. doi: 10.1158/1078-0432.CCR-10-2006 21088256

[13] Gummow BM Winnay JN Hammer GD. Convergence of Wnt signaling and steroidogenic factor-1 (SF-1) on transcription of the rat inhibin alpha gene. J Biol Chem (2003) 278:26572–9. doi: 10.1074/jbc.M212677200 12732619

[14] Botrugno OA Fayard E Annicotte JS Haby C Brennan T Wendling O. Synergy between LRH-1 and beta-catenin induces G1 cyclin-mediated cell proliferation. Mol Cell (2004) 15:499–509. doi: 10.1016/j.molcel.2004.07.009 15327767

[15] Schuijers J Mokry M Hatzis P Cuppen E Clevers H. Wnt-induced transcriptional activation is exclusively mediated by TCF/LEF. EMBO J (2014) 33:146–56. doi: 10.1002/embj.201385358 PMC3989608 24413017

[16] Lefèvre L Omeiri H Drougat L Hantel C Giraud M Val P. Combined transcriptome studies identify AFF3 as a mediator of the oncogenic effects of β-catenin in adrenocortical carcinoma. Oncogenesis (2015) 4:e161. doi: 10.1038/oncsis.2015.20 26214578 PMC4521181

[17] Mohan DR Borges KS Finco I LaPensee CR Rege J Solon AL. β-catenin-driven differentiation is a tissue-specific epigenetic vulnerability in adrenal cancer. Cancer Res (2023) 83:2123–41. doi: 10.1158/0008-5472.CAN-22-2712 PMC10330305 37129912

[18] Jalili V Afgan E Gu Q Clements D Blankenberg D Goecks J. The Galaxy platform for accessible, reproducible and collaborative biomedical analyses: 2020 update. Nucl Acids Res (2020) 48:W395–402. doi: 10.1093/nar/gkaa434 PMC7319590 32479607

[19] Bailey TL Johnson J Grant CE Noble WS. The MEME suite. Nucl Acids Res (2015) 43:W39–49. doi: 10.1093/nar/gkv416 PMC4489269 25953851

[20] Ferraz-de-Souza B Hudson-Davies RE Lin L Parnaik R Hubank M Dattani MT. Sterol O-acyltransferase 1 (SOAT1, ACAT) is a novel target of Steroidogenic Factor-1 (SF-1, NR5A1, Ad4BP) in the human adrenal. J Clin Endocrinol Metab (2011) 96:E663–668. doi: 10.1210/jc.2010-2021 PMC3124353 21239516

[21] Ehrlund A Jonsson P Vedin LL Williams C Gustafsson JÅ Treuter E. Knockdown of SF-1 and RNF31 affects components of steroidogenesis, TGFβ, and Wnt/β-catenin signaling in adrenocortical carcinoma cells. PloS One (2012) 7:e32080. doi: 10.1371/journal.pone.0032080 22427816 PMC3302881

[22] Bardou P Mariette J Escudié F Djemiel C Klopp C. jvenn: an interactive Venn diagram viewer. BMC Bioinf (2014) 15:293. doi: 10.1186/1471-2105-15-293 PMC4261873 25176396

[23] Zhou Y Zhou B Pache L Chang M Khodabakhshi AH Tanaseichuk O. Metascape provides a biologist-oriented resource for the analysis of systems-level datasets. Nat Commun (2019) 10:1523. doi: 10.1038/s41467-019-09234-6 30944313 PMC6447622

[24] Mizusaki H Kawabe K Mukai T Ariyoshi E Kasahara M Yoshioka H. Dax-1 (dosage-sensitive sex reversal-adrenal hypoplasia congenita critical region on the X chromosome, gene 1) gene transcription is regulated by wnt4 in the female developing gonad. Mol Endocrinol (2003) 17:507–19. doi: 10.1210/me.2002-0362 12554773

[25] Rena V Flores-Martín J Angeletti S Panzetta-Dutari GM Genti-Raimondi S. StarD7 gene expression in trophoblast cells: contribution of SF-1 and Wnt-beta-catenin signaling. Mol Endocrinol (2011) 25:1364–75. doi: 10.1210/me.2010-0503 PMC5417238 21622533

[26] Doghman M Cazareth J Lalli E. The T cell factor/beta-catenin antagonist PKF115-584 inhibits proliferation of adrenocortical carcinoma cells. J Clin Endocrinol Metab (2008) 93:3222–5. doi: 10.1210/jc.2008-0247 18544621

[27] Gaujoux S Hantel C Launay P Bonnet S Perlemoine K Lefèvre L. Silencing mutated β-catenin inhibits cell proliferation and stimulates apoptosis in the adrenocortical cancer cell line H295R. PloS One (2013) 8:e55743. doi: 10.1371/journal.pone.0055743 23409032 PMC3567123

[28] Leal LF Bueno AC Gomes DC Abduch R de Castro M Antonini SR. Inhibition of the Tcf/beta-catenin complex increases apoptosis and impairs adrenocortical tumor cell proliferation and adrenal steroidogenesis. Oncotarget (2015) 6:43016–32. doi: 10.18632/oncotarget.5513 PMC4767488 26515592

[29] Salomon A Keramidas M Maisin C Thomas M. Loss of β-catenin in adrenocortical cancer cells causes growth inhibition and reversal of epithelial-to-mesenchymal transition. Oncotarget (2015) 6:11421–33. doi: 10.18632/oncotarget.3222 PMC4484466 25823656

[30] Liu S Ding G Zhou Z Feng C. β-Catenin-driven adrenocortical carcinoma is characterized with immune exclusion. Onco Targets Ther (2018) 11:2029–36. doi: 10.2147/OTT.S159979 PMC5898592 29670378

[31] Ruggiero C Tamburello M Rossini E Zini S Durand N Cantini G. FSCN1 as a new druggable target in adrenocortical carcinoma. Int J Cancer (2023) 153:210–23. doi: 10.1002/ijc.34526 36971100

[32] Doghman M Cazareth J Douguet D Madoux F Hodder P Lalli E. Inhibition of adrenocortical carcinoma cell proliferation by Steroidogenic Factor-1 inverse agonists. J Clin Endocrinol Metab (2009) 94:2178–83. doi: 10.1210/jc.2008-2163 PMC2690427 19318454

[33] Zhang X Dong N Hu X. Wnt/β-catenin signaling inhibitors. Curr Top Med Chem (2023) 23:880–96. doi: 10.2174/1568026623666230303101810 36872364

