# Supplementary material for: A reappraisal of transcriptional regulation by NR5A1 and beta-catenin in adrenocortical carcinoma
Source: Front Endocrinol (Lausanne). 2023 Dec 8;14:1303332. doi: 10.3389/fendo.2023.1303332 (PMC10753177; doi:10.3389/fendo.2023.1303332)
Supplement: Supplementary file 1 [file DataSheet_1.pdf]

Supplementary Material

1 Supplementary Figures and Tables

1.1 Supplementary Figures

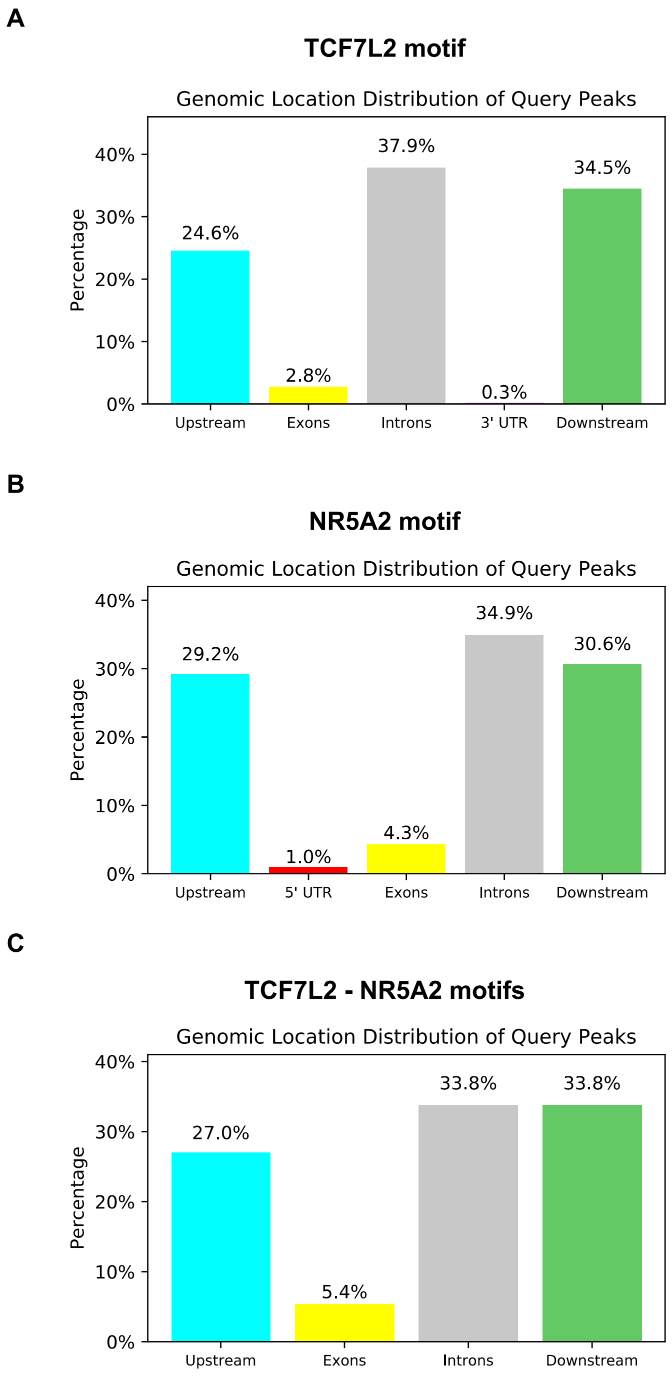

Supplementary Figure 1. Genomic distribution of intersect NR5A1 – beta-catenin peaks harbouring (**A**) TCF7L2 motifs, (**B**) NR5A2 motifs and (**C**) both motifs.

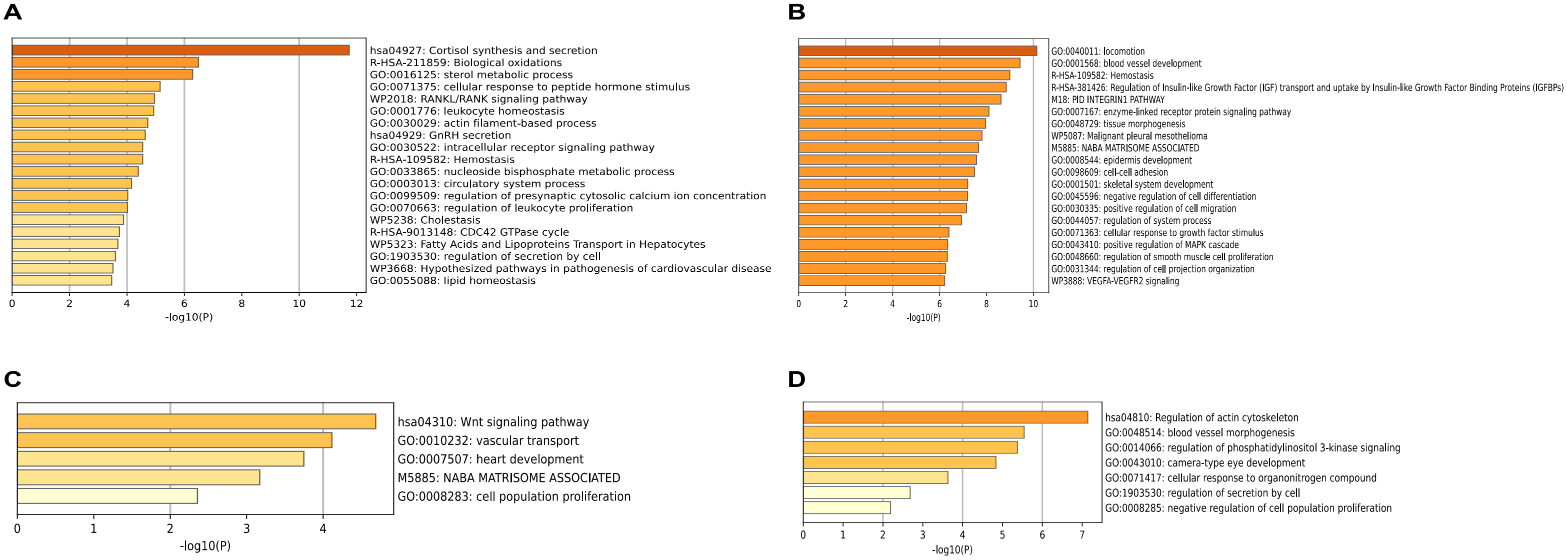

Supplementary Figure 2. Gene Ontology terms enriched in (**A**) NR5A1 positively regulated genes, (**B**) NR5A1 negatively regulated genes, (**C**) beta-catenin positively regulated genes and (**D**) beta-catenin negatively regulated genes.

2.2. Supplementary Tables

Table S1. List of NR5A1 ChIP peaks, beta-catenin ChIP peaks and NR5A1 - beta-catenin intersect ChIP peaks in H295R cells.

Table S2. List of TCF7L2 and NR5A2 motifs in the NR5A1 - beta-catenin intersect ChIP peaks in H295R cells.

Table S3. Overlap among gene sets differentially regulated after NR5A1/beta-catenin knockdown in H295R ACC cells.
